# Intestinal Parasitic Infections in Mental Retardation Center of Bandar Abbas, Southern Iran

**Published:** 2019

**Authors:** Vahedeh MOHAMMADI-MESKIN, Yaghoob HAMEDI, Mehrgan HEYDARI-HENGAMI, Ebrahim EFTEKHAR, Jebreil SHAMSEDDIN, Khojasteh SHARIFI-SARASIABI

**Affiliations:** 1. Department of Parasitology and Mycology, Faculty of Medicine, Hormozgan University of Medical Sciences, Bandar Abbas, Iran; 2. Infectious and Tropical Diseases Research Center, Hormozgan Health Institute, Hormozgan University of Medical Sciences, Bandar Abbas, Iran; 3. Molecular Medicine Research Center, Hormozgan Health Institute, Hormozgan University of Medical Sciences, Bandar Abbas, Iran

**Keywords:** Mentally disabled, *Strongyloides stercoralis*, Intestinal parasites, Iran

## Abstract

**Background:**

The present study aimed to evaluate the prevalence of intestinal parasitic infection among mentally retarded individuals and the staff of the center in Bandar Abbas, south of Iran.

**Methods:**

This cross-sectional study was conducted in central institution for mentally retarded in Bandar Abbas, Hormozgan Province, from 2016 to 2017. A triple fecal specimen was collected from each one of the 163 participants and were evaluated using wet mount and formalin-ethyl acetate methods. Trichrome and Ziehl-Neelsen staining were used to confirm suspected cases of protozoa. As well as Baermann and Harada-Mori techniques and agar plate culture were implemented to diagnosis of *Strongyloides stercoralis*.

**Results:**

Overall, 163 subjects were examined including 126 mentally retarded individuals and 37 personnel. Ninety (55.2%) cases of participants were infected with at least one of the intestinal parasites, 69 (54.7%) of mentally retarded and 21 (56.7%) of personnel. Twenty-six mentally retarded individuals were infected with *S. stercoralis* (20.6%), *Blastocystis hominis* 30.2%, *Entamoeba coli 25.4%, Giardia lamblia* 5.6%, *Enterobius vermicularis* 1.6%, *Hymenolepis nana* 0.8% and *Iodamoeba butschlii* 1.6%*.* Twelve staff (32.4%) harbored *B. hominis, E. coli 27%, S. stercoralis* 2.7%, *G. lamblia 10.8%, H. nana* 2.7% and *Endolimax nana* 2.7%.

**Conclusion:**

High rate of intestinal parasites particularly, *S. stercoralis* compared to the most recent studies of general population. Therefore, regular screening and tracking the positive cases, disinfection of the living environment, training and financing of the staff, increasing the number of the workers, recruiting of professionals and trained personnel in these centers are suggested.

## Introduction

The contamination of water and food with human wastes and inadequate health systems are the main causes of fecal-oral transmission of intestinal parasitic infections. There is a meaningful relationship between parasitic infections and the health behavior of people in the community (1). The intestinal pathogens excreting through the feces cause the contamination of the fingers and consequently the foods) (2). In addition, there is a direct relationship between parasitic infections and socioeconomic status of the community (3). Many non-pathogenic intestinal parasites have been observed in the stool sample of mentally retarded institutes (4, 5). Such environments due to the large population and life-long closed contact particularly in those with low sanitary level transmit the intestinal parasites (6). Parasitic infections particularly *Strongyloides stercoralis*is are major problems in rehabilitation centers (7, 8). It can infect humans for years because it is unique among parasites due to having natural internal cycle, without further exposure to contaminated soils or intermediate hosts (9). The infection has a chronic nature due to autoinfection without clinical symptoms ignored for years (10, 11).

In the present study, unlike the previous study (8), the staff of the center in addition to the mentally retarded individuals were evaluated. Furthermore, *S. stercoralis*is was assessed with specific methods such as agar plate culture, Baermann (12) and Harada-Mori (13) techniques. The present study aimed to evaluate and tracking the prevalence of intestinal parasitic infection among mentally retarded individuals and the staff of the central institute in Bandar Abbas, south of Iran.

## Materials and Methods

### Study population and sample collection

This cross-sectional study was conducted in the central institution for mentally retarded individuals in Bandar Abbas, Hormozgan Province, from Aug 2016 to Sep 2017. Almost all mental retarded individuals in the province, including children and adults, are maintained at this center. Bandar Abbas is situated on a flat area with an average altitude of 9 m (30 ft.) above sea level and a population of approximately 450,000 people (14). Maximum temperature in summers can reach 49 °C (120 °F) while in winters the minimum temperature may drop to 5 °C (41 °F). The annual rainfall is around 170 mm (6.7 in) and the average relative humidity is 65% (15).

Out of 150 mentally retarded individuals, 126, and of 55 employees and workers 37 individuals were evaluated. For each subject, three fresh fecal specimens were collected in a clean stool cup every other day. Each container was labeled and immediately sent to the parasitology laboratory at the School of Medicine of Hormozgan University of Medical Sciences (HUMS). A questionnaire for the mentally retarded patients was designed based on demographic data such as age, gender, the rate of physical disability and the history of their residence in the institute, and for the staff the years they were working, as well as educational level. The staff trained on sample collection, such as the time of sampling and the amount of sample.

### Statistical analysis

Statistical analysis of data was done using SPSS (version 20, Chicago, IL, USA) software. *P*<0.05 was considered as a meaningful standard. The prevalence of intestinal parasites evaluated with descriptive statistics, while the relationship between the categorical variables and presence of intestinal parasites assessed by the chi-square test.

### Ethical Considerations

The study protocol was approved by the Infectious and Tropical Diseases Research Center, Hormozgan Health Institute, (HUMS), Bandar Abbas, Iran with the code of ethics, HUMS REC 1395.54. All relevant authorities and headmasters of the rehabilitation center were informed about the purpose and procedure of the study. Written informed consent was obtained from the parents or the legal guardians of the patients and from the staffs, prior to study onset.

### Stool examination

All specimens evaluated via wet mount and formalin-ethyl acetate concentration techniques. Trichrome and Ziehl-Neelsen staining were used for accurate confirmation of protozoa. In order to diagnose *S. stercoralis,* agar plate culture, Baermann, and Harada-Morri techniques were used.

## Results

Out of 163 participants, 126 mentally retarded individuals and 37 staff were examined for intestinal parasitic infections. The mean age of the study was 25.9±11.5 and 40.7±8.6 yr for mentally retarded and staff respectively. The populations’ ages ranged from 4 to 66 yr old. Ninety (55.2%) cases of participants were infected with at least one of the intestinal parasites (Table 1).

**Table 1:** Prevalence of intestinal parasitic infections based on age distribution among 163 residents of the rehabilitation center and personnel in Bandar Abbas, southern Iran, 2017

***Age group (yr)***	***Subjects (%)***	***Infected (%)***
10 <	8 (4.9)	4 (50)*
11–20	35 (21.5)	20 (57.1)
21–30	49 (30)	21 (42.9)
31–40	39 (23.9)	23 (59)
40 >	32 (19.6)	22 (68.7)
Total	163	90 (55.2)

*P*-value= 0.239

As shown in Table 2, the most common protozoan species were *Blastocystis hominis* (n= 50, 30.7%) and *Entamoeba coli* (n= 42, 25.8%) and the most common helminthes was *S. stercoralis* (n=27, 16.6%). *S. stercoralis* found in only one of the 37 personnel (2.7%).

**Table 2:** Prevalence of intestinal parasitic infections among 163 residents of the rehabilitation center in Bandar Abbas, southern Iran, 2017

***Intestinal parasites***	***Mentally retarded n=126 (%)***	***Personnel n=37 (%)***	***Total n=163 (%)***
*Blastocystis hominis*	38 (30.2)	12 (32.4)	50 (30.7)
*Entamoeba coli*	32 (25.4)	10 (27)	42 (25.8)
*Strongyloides stercoralis*	26 (20.6)	1 (2.7)	27 (16.6)
*Giardia lamblia*	7 (5.6)	4 (10.8)	11 (6.7)
*Hymenolepis nana*	1 (0.8)	1 (2.7)	2 (1.2)
*Endolimax nana*	-	1 (2.7)	1 (0.6)
*Enterobius vermicularis*	2 (1.6)	-	2 (1.2)
*Iodamoeba butschlii*	2 (1.6)	-	2 (1.2)

Overall, there was a statistically significant difference in prevalence of intestinal parasites in mentally retarded individuals based on gender (*P*=0.037) and the rate of physical disability (*P*=0.044) but no difference was seen based on age and the history of residency in the institute (Table 3).

**Table 3:** The relationship between prevalence of intestinal parasitic infections and certain assumed risk factors among 126 mentally retarded individuals in Bandar Abbas, southern Iran, 2017

***Variable***	***Frequency (%)***	***Parasitic infection (%)***	***P-value***
Gender			
Male	63 (50)	40 (58)	0.037
Female	63 (50)	29 (42)	
Age group (yr)			
0–15	24 (19)	11 (15.9)	0.447
16–25	44 (34.9)	23 (33.3)	
>26	58 (46)	35 (50.7)	
The rate of physical disability			
Intermediate	12 (9.5)	8 (11.6)	0.044
Sever	45 (35.7)	18 (26.1)	
Too Sever	69 (54.8)	43 (62.3)	
History of residency (yr)			
<10	73 (57.9)	36 (52.2)	0.349
11–20	22 (17.5)	14 (20.3)	
>21	31 (24.6)	19 (27.5)	

There was no significant difference in the rate of intestinal parasites infection in personnel based on gender, age, the level of education, the work experience and the direct contact with the mentally retarded individuals (Table 4).

**Table 4:** The relationship between prevalence of intestinal parasitic infections and certain assumed risk factors among 37 personnel in Bandar Abbas, southern Iran, 2017

***Variable***	***Frequency (%)***	***Parasitic infection (%)***	***P-value***	***OR***
Gender				
Male	12 (32.4)	7 (33.3)	0.589	1.1[0.273,4.430]
Female	25 (67.6)	14 (66.7)		
Age group (yr)				
20–30	6 (16.2)	3 (14.3)	0.351	
31–40	10 (27.0)	4 (19.0)		
>41	21 (56.8)	14 (66.7)		
Educational level				
Reading & writing	25 (67.6)	15 (71.4)	0.411	1.5[0.375,5.998]
Formal education	12 (32.4)	6 (28.6)		
Work experience				
1–10	21 (56.8)	9 (42.9)	0.140	
11–20	13 (35.1)	10 (47.6)		
21–30	3 (8.1)	2 (9.5)		
Direct contact with the patient				
Yes	30 (81.1)	14 (66.7)	0.053	0.133[0.015,1.226]
No	7 (18.9)	7 (33.3)		

## Discussion

Considering the whole employed methods in the study, prevalence of intestinal parasitic infections was 55.2% where the infection was significantly higher than the other populations in this area (2, 12). However, is consistent with the studies that conducted in the mentally retarded institutes of the other countries, such as Egypt (43.5%), Puerto Rico (52.3%) and Italy (53.8%) (16–18) and Kashan (64.8%) in central part of Iran (19).

The lower rates of parasitic infections were seen in rehabilitation centers of northern Iran, Mazandaran Province 5.15%, Qaemshahr 26.2%, Rasht 29.5% and Urmia 20.4% (4–5, 10, 20).

*S. stercoralis* (16.6%) was the most pathogen helminthes amongst the residents of the mentally retarded institute in Bandar Abbas, south of Iran. The prevalence of this parasite was significantly higher among mentally retarded individuals (n= 26, 20.6%) compared to the staff (n=1, 2.7%) of the center. In Mazandaran and Qaemshahr, (5, 20) the other intestinal parasites except *S. stercoralis* were observed, while in the surveys carried in Rasht (10) and Kashan (19) the rates of *S. stercoralis* were 1.2% and 0.4%, respectively. The studies in other countries such as Italy and Philippines showed a very low incidence of this parasite (17, 21). Prevalence of *S. stercoralis* in the mentally retarded population (20.6%) compared to the pervious study, 17.3% (8) revealed the higher incidence perhaps it was due to the use of special methods for detection of the parasite. However, the prevalence of *S. stercoralis* at this center was not much different from the previous study in this institute, which could reveal the challenges in this center requiring great effort to resolve it.

*S. stercoralis* is a soil-transmitted helminth, but in this study, none of the other soil-transmitted helminthes was seen in this community. The absence of the other intestinal worms in these individuals may be due to the long process of maturation of embryonated egg (7). The high prevalence of *S. stercoralis* among the mentally retarded should not be considered as a sudden infection, but these are due to the previous, chronic, accumulated and untreated infections. The high prevalence of this parasite in the previous study (8) is the reason for this claim. High rates of intestinal parasitic infections among the mentally retarded patients can be considered as a great risk for transmission to the other people. Considering the patients are screened and treated regularly, their inability to respect personal hygiene is the reason for the return and survival of the parasite because of internal and external autoinfection (22). Bandar Abbas has a warm and relatively humid climate (8). Therefore, in this condition, the larvae excreted from the stool adhere to the skin or hairs of the anal region, turn into infectious larva and enter the body through the skin (7). North of Iran also has a humid climate, and in addition, the parasite is endemic in these areas (23), but the prevalence of this parasite in mentally retarded institute in these area is low (5, 10, 20). Hence, the reason for the high prevalence of this parasite in this center should be investigated. The other reasons for strongyloidiasis comprise licking or sucking the fingers or objects infected with fecal infected materials, which lead to penetration the larva through oral mucosa (7). However, there are the same conditions in other centers; researches should be done to determine the other transmission pathways for this parasite. The existing risk factors are required to be determined carefully.

In general, the prevalence of intestinal parasites in the staff was not significantly different compared to the mentally retarded population, but about *S. stercoralis* drastically significant. *S. stercoralis* was found in only one of the 37 personnel who work in close contact with the mentally retarded patients in the center.

We expected to see the high rates of directly transmitted helminthes, particularly *H. nana* and *E. vermicularis* however, similar to the other studies (4–6, 10) the low rate of these helminthes were seen. Of course, *E. vermicularis* was merely evaluated by the stool exam not the scotch tape procedure.

*B. hominis* and *Entamoeba coli* were the other intestinal parasites in this institute with a prevalence of 30.2% and 25.4% respectively. Compared to the other studies in Mazandaran Province, (5) Rasht (10), and Urmia in Iran (4) the prevalence of *E. coli* and *B. hominis* were significantly high, but consistent with the infection rate of the other intestinal protozoa including *E. nana* and *Iodamoeba butschlii.* Although identification of non-pathogen parasites is not important, it usually indicates fecal-oral contamination.

*G. lamblia*, one of the other pathogen parasites, compared to the previous study (8) 2.3%, has been increased to 5.6%, which is consistent to another study in Egypt (6) and in Urmia, Iran (4). Since this pathogen transmits by using unhealthy drinking water, contaminated and non-sanitary food and raw vegetables, so were higher in the staff (10.8%) compared to the mentally retarded (5.6%) due to preparation of meal by the staff. The prevalence of *G. lamblia* in the food handlers of Bandar Abbas (6.8%) is the reason for this claim (2). However, S. *stercoralis*, which is different from the other parasites due to the nature of its transmission to human, is more common in these people.

Unlike the studies conducted in Egypt (6) and Kashan in Iran (19) our finding revealed no cases of *E. histolytica/E. dispar*, and *Dientamoeba fragilis* or other opportunistic pathogens such as *Cryptosporidium* sp., and *Cyclospora Cayetanensis*.

The age of the patients and the history of residency in the center affected the frequency of intestinal parasites; contrary to our understanding, it became evident that the sex of the patients was an effective factor. This is consistent with a study conducted in Cambodia that gender has a positive role in dealing with this parasite (24), however, it was inconsistent with the study in Egypt (6) that the sex did not affect the level of infected individuals. Of course, the rate of infection in older age groups was much higher hence and was not significant.

As we expected the rate of physical ability of patients, due to its lack of capacity in complying with personal hygiene affects the frequency of intestinal parasites. It was, however, found that their physical disability was affected.

On part of the staff working in the center as well as the frequency of intestinal parasites, we expected that direct contact with the patient and the work experience would play important role, as shown in Table 4, direct contact with the patient was somewhat influential, and however this effect was not significant. It was consistent with the study (7) which showed no contamination in any of the personnel involved with the patients.

## Conclusion

Intestinal parasites are still a major problem for the mentally retarded individuals in Bandar Abbas where S. *stercoralis* is particularly considered another problem in this rehabilitation center. The dramatical decrease of intestinal parasites in population in recent years might reflect the improvement in the living conditions, sanitary facilities and increased levels of literacy but the decline is not necessarily seen in mentally retarded institution. They do not have an adequate understanding of individual health and intestinal parasites. Therefore, in spite of all the efforts of the officials of the center and the staff, they infect themselves and the other roommates. High rates of *S. stercoralis* indicate the challenges in this center and the problems they are tackling. It seems the researches should be carried out to ensure that there are the other transmission routes for these individuals in spite of endless efforts of the staff. Measures should be taken, including regular screening and follow up of positive cases, mass treatment, disinfection of the living environment, staff training and financing, increasing the number of worker, recruiting professional and trained personnel in these centers.

## References

[1] Alyousefi NA Mahdy MA Mahmud R Lim YA Factors associated with high prevalence of intestinal protozoan infections among patients in Sana’a City, Yemen. PLoS One. 2011;6(7):e22044. 21789210 10.1371/journal.pone.0022044 PMC3138770

[2] Heydari-Hengami M Hamedi Y Najafi-Asl M Sarifi-Sarasiabi K Prevalence of Intestinal Parasites in Food Handlers of Bandar Abbas, Southern Iran. Iran J Public Health. 2018;47(1):111–118. 29318125 PMC5756585

[3] Kia E Hosseini M Nilforoushan M Meamar A Rezaeian M Study of intestinal protozoan parasites in rural inhabitants of Mazandaran province, Northern Iran. Iran J Parasitol. 2008;3(1):21–5.

[4] Tappeh KH Mohammadzadeh H Rahim RN Prevalence of intestinal parasitic infections among mentally disabled children and adults of Urmia, Iran. Iran J Parasitol. 2010;5(2):60–4. 22347245 PMC3279829

[5] Sharif M Daryani A Asgarian F Nasrolahei M Intestinal parasitic infections among intellectual disability children in rehabilitation centers of northern Iran. Res Dev Disabil. 2010;31(4):924–8. 20363588 10.1016/j.ridd.2010.03.001

[6] Shehata AI Hassanein F Intestinal parasitic infections among mentally handicapped individuals in Alexandria, Egypt. Ann Parasitol. 2015;61(4):275–81. 26878626 10.17420/ap6104.19

[7] Yoeli M Most H Berman HH Tesse B I. The problem of strongyloidiasis among the mentally retarded in institutions. Trans R Soc Trop Med Hyg. 1963; 57:336–45. 14062269 10.1016/0035-9203(63)90096-8

[8] Shokri A Sarasiabi KS Teshnizi SH Mahmoodi H Prevalence of *Strongyloides stercoralis* and other intestinal parasitic infections among mentally retarded residents in central institution of southern Iran. Asian Pac J Trop Biomed. 2012;2(2):88–91. 23569874 10.1016/S2221-1691(11)60198-6 PMC3609260

[9] Kia EB Rahimi HR Mirhendi H A case of fatal strongyloidiasis in a patient with chronic lymphocytic leukemia and molecular characterization of the isolate. Korean J Parasitol. 2008;46(4):261–263. 19127333 10.3347/kjp.2008.46.4.261 PMC2612612

[10] Saeidinia A Tavakoli I Naghipour MR Prevalence of *Strongyloides stercoralis* and Other Intestinal Parasites among Institutionalized Mentally Disabled Individuals in Rasht, Northern Iran. Iran J Parasitol. 2016;11(4):527–33. 28127364 PMC5251181

[11] Meamar A Rezaian M Mohraz M *Strongyloides stercoralis* hyper-infection syndrome in HIV+/AIDS patients in Iran. Parasitol Res. 2007;101(3):663–5. 17401580 10.1007/s00436-007-0531-x

[12] Khieu V Schär F Marti H Diagnosis, treatment and risk factors of *Strongyloides stercoralis* in schoolchildren in Cambodia. PLoS Negl Trop Dis. 2013;7(2):e2035. 23409200 10.1371/journal.pntd.0002035 PMC3566990

[13] Requena-Méndez A Chiodini P Bisoffi Z The laboratory diagnosis and follow up of strongyloidiasis: a systematic review. PLoS Negl Trop Dis. 2013;7(1):e2002. 23350004 10.1371/journal.pntd.0002002 PMC3547839

[14] Turki H Hamedi Y Heidari-Hengami M Prevalence of intestinal parasitic infection among primary school children in southern Iran. J Parasit Dis. 2017;41(3):659–65. 28848255 10.1007/s12639-016-0862-6 PMC5555908

[15] Farshidi H Zare S Eghbal Eftekhaari T Nikbakht Mobarake F Association of Climate with Acute Myocardial Infarction Hospitalizations. Hormozgan Medical Journal. 2017; 21(2):77–85.

[16] Sirivichayakul C Pojjaroen-anant C Wisetsing P Prevalence of intestinal parasitic infection among Thai people with mental handicaps. Southeast Asian J Trop Med Public Health. 2003; 34(2): 259–63. 12971546

[17] Ferrer-Rodríguez I Kozek WJ Prevalence of intestinal parasitoses among patients and staff of an institution for the mentally retarded. J Parasitol Vector Biol. 2011;3(5):69–74.

[18] Giacometti A Cirioni O Balducci M Epidemiologic features of intestinal parasitic infections in Italian mental institutions. Eur J Epidemiol. 1997;13(7):825–30. 9384273 10.1023/a:1007306630301

[19] Rasti S Arbabi M Hooshyar H High prevalence of *Entamoeba histolytica* and *Enterobius vermicularis* among elderly and mentally retarded residence in Golabchi center, Kashan, Iran 2006–2007. Jundishapur J Microbiol. 2012;5(4):585–9.

[20] Soleymani E Davoodi L Azami D The Prevalence of Intestinal Parasitic Infections among the Mentally Retarded Patients in Lamook Rehabilitation Center of Qaemshahr, Mazandaran Province, 2015. Tabari J Prev Med. 2016; 2(1): 1–5

[21] Cross J Banzon T Wheeling C Biomedical survey in North Samar Province, Philippine Islands. Southeast Asian J Trop Med Public Health. 1977;8(4):464–75. 614707

[22] Teixeira M Inês EdJ Pacheco F Asymptomatic *Strongyloides stercoralis* hyperinfection in an alcoholic patient with intense anemia. J Parasitol. 2010;96(4):833–5. 20738204 10.1645/GE-2358.1

[23] Daryani A Sharif M Nasrolahei M Epidemiological survey of the prevalence of intestinal parasites among schoolchildren in Sari, northern Iran. Trans R Soc Trop Med Hyg. 2012;106(8):455–9. 22703897 10.1016/j.trstmh.2012.05.010

[24] Khieu V Schär F Marti H Prevalence and risk factors of *Strongyloides stercoralis* in Takeo Province, Cambodia. Parasit Vectors. 2014; 7:221. 24886763 10.1186/1756-3305-7-221 PMC4029906

